# Supplementary material for: Sialic acid O-acetylation patterns and glycosidic linkage type determination by ion mobility-mass spectrometry
Source: Nat Commun. 2023 Oct 25;14:6795. doi: 10.1038/s41467-023-42575-x (PMC10600165; doi:10.1038/s41467-023-42575-x)
Supplement: Supplementary file 1 — Supplementary Information [file 41467_2023_42575_MOESM1_ESM.pdf]

Supplementary Information

Sialic Acid *O*-Acetylation Patterns and Glycosidic Linkage Type Determination by Ion Mobility-Mass Spectrometry

Gaël M. Vos[1+], Kevin C. Hooijschuur[1+], Zeshi J. Li[1], John Fjeldsted[2], Christian Klein[2], Robert P. de Vries[1], Javier Sastre Toraño[1*], Geert-Jan Boons[1,3,4*]

[1] Department of Chemical Biology and Drug Discovery, Utrecht Institute for Pharmaceutical Sciences, Utrecht University, Universiteitsweg 99, 3584 CG Utrecht, The Netherlands

[2] Agilent Technologies, Santa Clara, CA 95051, United States

[3] Bijvoet center for Biomolecular Research, Utrecht University, 3584 CG Utrecht, The Netherlands

[4] Complex Carbohydrate Research Center and Department of Chemistry, University of Georgia, 315 Riverbend Road, Athens, GA 30602, United States

Table of Contents

Supplementary Note 1**:** Enzymatic pathways and observations for structural assumptions of *N*-glycan structures 3

Supplementary Figure 1: MS fragmentation spectrum of the most abundant structure in the rear tracheal tissue. 4

Supplementary Figure 2: Venn diagram displaying glycan compositions that were shared across the three nasal tissue samples, or unique to a single sample. ..5

Supplementary Figure 3: Glycan microarrays of the HA ectodomains if human H3N2. 6

Supplementary Table 1: Supplementary Glycan Microarray Information, Based on MIRAGE Guidelines..........7

Supplementary Table 2: Compound map of the Glycan Microarray...........10

Supplementary Note 1: Enzymatic pathways and observations for structural assumptions of *N*-glycan structures

[1]: Core fucosylation was assumed due to an abundance of the $Y_{1\alpha}$ fragment (Fuc-GlcNAc-Procainamide) and Y_2 fragment (GlcNAc-(Fuc)-GlcNAc-Procainamide) fragments and the general absence of fragments that indicate antenna fucosylation (see figure S1 below).

[2]: After glycosylation of the *N*-glycan site, trimming of the 9-mannose *N*-glycan yields the branched trimannose on the α1-6Man-linked branch of the *N*-glycan core. These are only removed after installation of the GlcNAc on the α1-3Man-linked branch by MGAT1 (Stanley et al., 2022, *Essentials of Glycobiology 4th ed.*, Ch9: *N*-glycans). Further assumptions cannot be made, as the order of mannosidase cleavage is ambiguous (Roth, 2002, *Chem. Rev.* 102, 2, 285–304), and multiple isomeric variants have been observed on glycoproteins (Wei et al., 2020, *Anal. Chem.* 92, 1, 782–791).

[3]: Although not depicted here, the α2,6 monosialylated structures predominantly feature the sialic acid on the α1-3Man-linked branch of the complex *N*-glycan, as ST6Gal1 has a more than tenfold preference for the α1-3Man-linked branch (Barb et al., 2009, *Biochemistry*, 48, 9705-9707 and Barb&Prestegard, 2011, *Nat. Chem. Biol.*, 7, 147-153). But since a small amount of α2,3-linked sialosides was observed, this assumption cannot be made with full certainty.

[4]: As sialic acids are essentialy always terminal, preventing further extention of the *N*-glycan antennae, the position of the sialic acids and other monosaccharides can be assumed here (Lewis et al., 2022, *Essentials of Glycobiology 4th ed.*, Ch 15: Sialic acids and other nonulosonic acids).

[5]: The LacNAc to the left of the accolade could be an MGAT4 or MGAT5 product, dictating its position on the α1-3Man-linked branch or α1-6Man-linked branch respectively (Stanley et al., 2022, *Essentials of Glycobiology 4th ed.*, Ch9: *N*-glycans).

[6]: The alpha-Gal epitope features a terminal galactose that is always α1,3-linked to the LacNAc, so we can assume its linkage (Stanley et al. *Essentials of Glycobiology 4th ed.*, Ch 14: Structures common to different glycans). We observed $Hex_2HexNAc$ fragment ions (*m/z* 528.1923, Fig. S1), which we attribute to alpha-Gal resulting of a single cleavage, whereasas an internal $Hex_2HexNAc$ fragment is possible but unlikely due to the necessity of multiple cleavages.

[7]: Both a hybrid structure and a complex biantennary glycan are possible here.

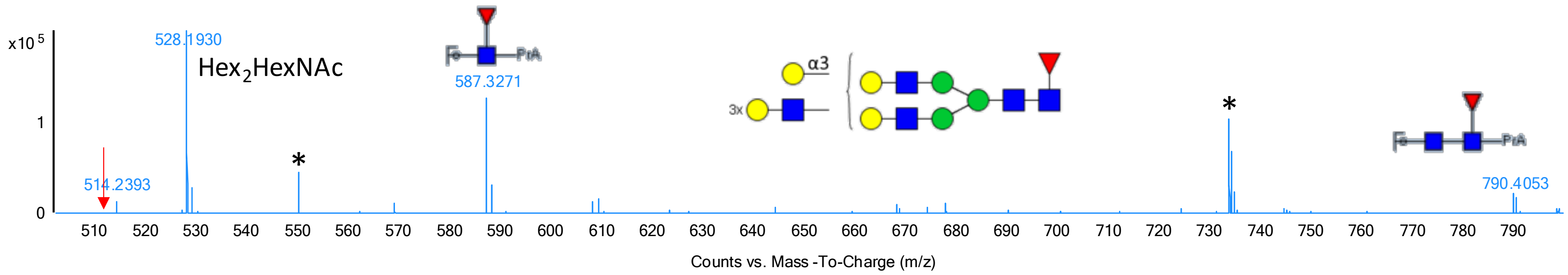

Supplementary Figure 1: MS fragmentation spectrum of the most abundant structure in the rear tracheal tissue.

Y1α and Y2 fragments (Fuc-GlcNAc-Procainamide and GlcNAc-(Fuc)-GlcNAc-Procainamide) are identified and absence of antenna fucosylation (Fuc-LacNAc, m/z 512.1974) is indicated by a red arrow. The Hex2HexNAc fragment points toward the alpha-Gal epitope, see [6] above. *unassigned fragments or coeluting species

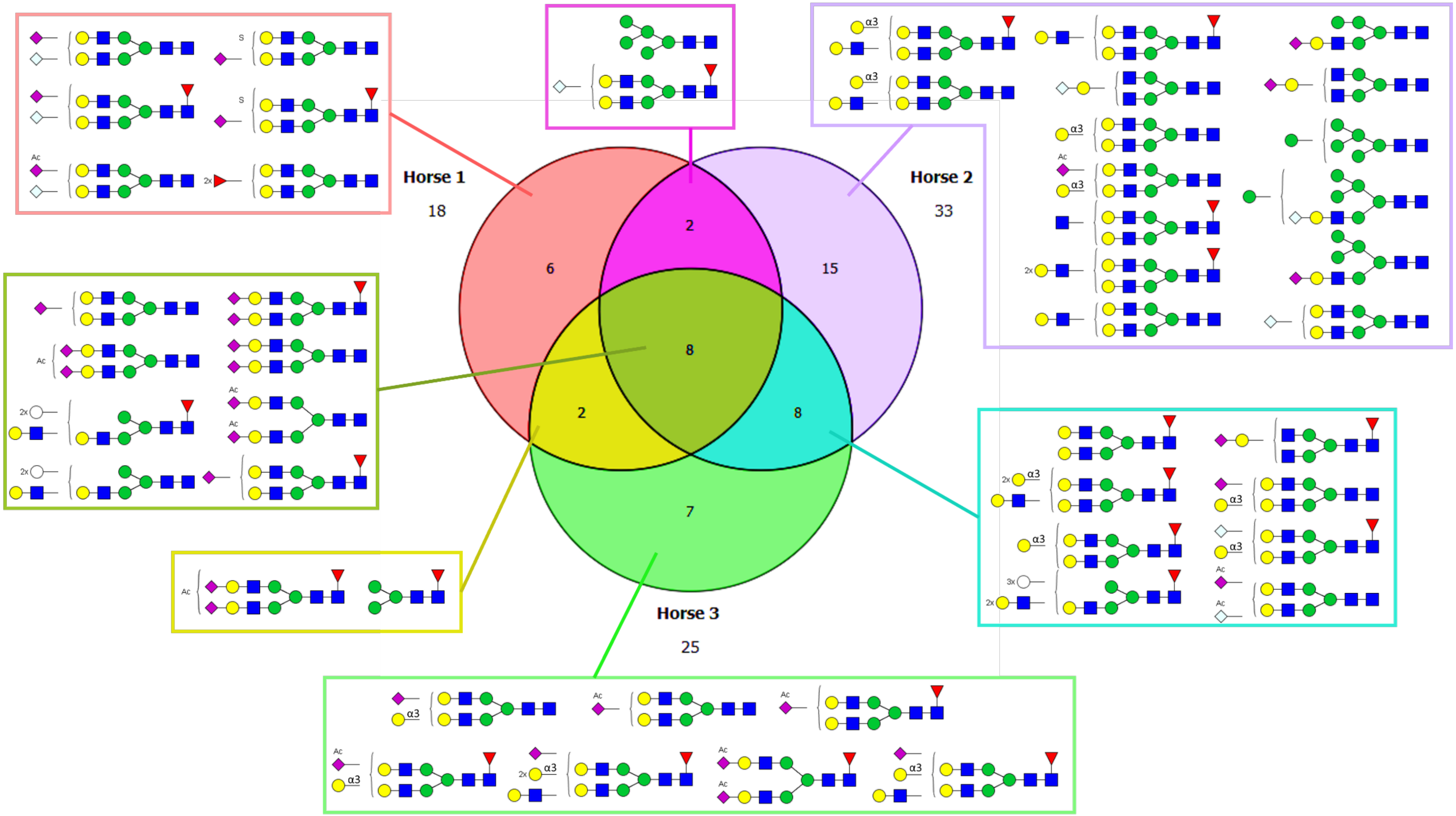

Supplementary Figure 2: Venn diagram displaying glycan compositions that were shared across the three nasal tissue samples, or unique to a single sample.

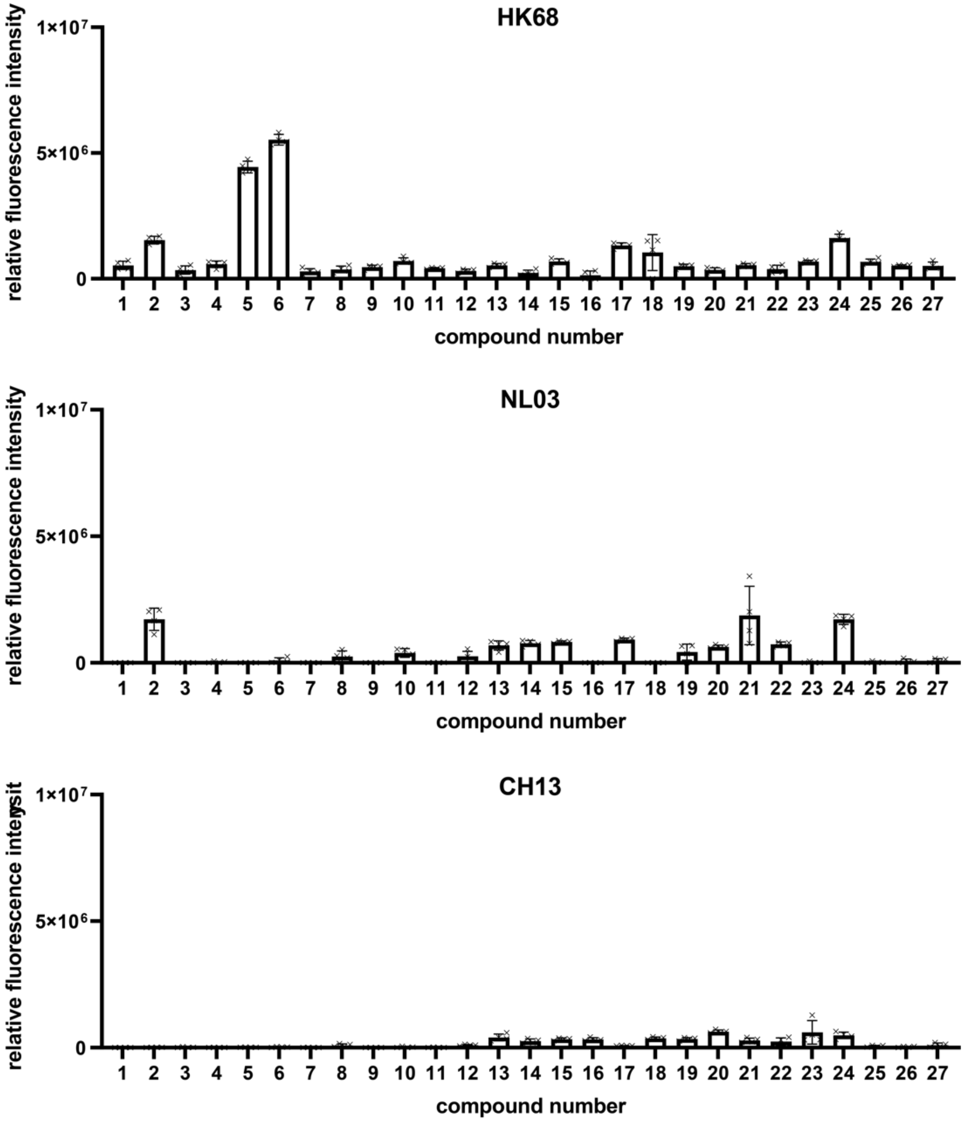

Supplementary Figure 3: Glycan microarrays of the HA ectodomains if human H3N2.

Glycan microarray analysis (mean±SD, n=4). Top: HA ectodomain of HK68 (A/Hong kong/1/68 H3N2, Genbank accession no. AF358177). Middle: HA ectodomain of NL03 (A/Netherlands/213/03 H3N2, Genbank accession no. AY661035). Bottom: HA ectodomain of CH13 (A/Switzerland/9715293/13 H3N2, Genbank accession no. AU46905.1). The compound number refers to the structures in Fig. 1. Source data are provided as a Source Data file.

Supplementary Table 1: Supplementary Glycan Microarray Information, Based on MIRAGE Guidelines (doi:10.3762/mirage.3)

	**Description***
**1. Sample: Glycan Binding Sample**	
Description of Sample	Figure 5<br>A/Equine/New York/49/73<br>A/Equine/Miami/1/1963<br><br>Supplementary Figure 3<br>A/Hong kong/1/68<br>A/Netherlands/213/03<br>A/Switzerland/9715293/13<br>HA-encoding cDNA was cloned into the pCD5 expression vector as described previously. The resulting expression vector encodes an HA protein containing a heterologous signal peptide, a C-terminal trimerization domain (GCN4), sfGFP, and a double strep-tag, and lacking the transmembrane and cytoplasmic domains.<br>The HA proteins were expressed in HEK293S GnT1(−) cells, purified from the cell culture supernatants using streptactin beads, and quantified. (Nemanichvili, N., Tomris, I., Turner, H.L., McBride, R., Grant, O.C., van der Woude, R., Aldosari, M.H., Pieters, R.J., Woods, R.J., Paulson, J.C., Boons, G.J., Ward, A.B., Verheije, M.H. & de Vries, R.P. Fluorescent trimeric hemagglutinins reveal multivalent receptor binding properties. J. Mol. Biol. 431, 842-856 (2019). [https://doi: 10.1016/j.jmb.2018.12.014].
Assay protocol	HA (50 µg/mL) were precomplexed with anti-streptag-Alexa647 and goat-anti mouse-Alexa647 in 50 µL for 15 min on ice and incubated on the array for 90 min at room temperature.

2. Glycan Library	
Glycan description for defined glycans	Li, Z., Lang, Y., Liu, L., Bunyatov, M.I., Sarmiento, A.I., de Groot, R.J. & Boons, G.J. Synthetic O-acetylated sialosides facilitate functional receptor identification for human respiratory viruses. Nat. Chem. 13, 496-503 (2021). [https://doi.org/10.1038/s41557-021-00655-9].
1. **3. Printing Surface; e.g., Microarray Slide**	
Description of surface	Streptavidin-coated glass slides (SuperStreptavidin Microarray Substrate Slides)
Manufacturer	ArrayIt Inc
Covalent Immobilization	-
**4. Arrayer (Printer)**	
Description of Arrayer	Non-contact microarray printer, sciFLEXARRAYER S3, Scienion Inc.
Dispensing mechanism	Non-contact, one nozzle
Glycan deposition	400 pL of 100 µM, 40 fmol, 6 replicates
Printing conditions	100 µM printing concentration, 20 °C, 50% humidity, blocking with TMS binding buffer (20 mM TRIS·HCl, pH 7, 150 mM NaCl, 2mM $CaCl_2$, 2 mM $MgCl_2$, 0.05% Tween-20 and 1% BSA) for 1 h at 4 °C prior to use. Slides were rinsed with DI water.
2. **5. Glycan Microarray with "Map"**	
Array layout	24 subarrays were printed per slide.

Glycan identification and quality control	For identification and quality control see Li, Z., Lang, Y., Liu, L., Bunyatov, M.I., Sarmiento, A.I., de Groot, R.J. & Boons, G.J. Synthetic O-acetylated sialosides facilitate functional receptor identification for human respiratory viruses. Nat. Chem. 13, 496-503 (2021). [https://doi.org/10.1038/s41557-021-00655-9].
**3. 6. Detector and Data Processing**	
Scanning hardware	Innopsys Innoscan 7200
Scanner settings	Iterative scans at 647 nm, laserpower at 1%, 5%, 10%, 50% and 100% (647 nm).<br>The lower laserpowers were used to avoid overexposure.
Image analysis software	GenePix Pro 7 software
Data processing	Fluorescent intensity from the images, highest and lowest value removed from 6 replicates, total intensities are plotted as mean +/- SD.
**7. Glycan Microarray Data Presentation**	
Data presentation	Figure 5 and Supplementary Figure 3
4. **8. Interpretation and Conclusion from Microarray Data**	
Data interpretation	Images were analyzed using GenePix Pro 7 software. The data were further processed with Microsoft Excel and plotted with GraphPad Prism 7.<br>The script for Microsoft Excel Macro for batch processing glycan microarray data is uploaded to<br>https://github.com/enthalpyliu/carbohydrate-microarray-processing. https://doi.org/10.5281/zenodo.5146251
Conclusions	Equine H3N8 and H7N7 viruses bind 4-*O*-acetylated α2-3 linked sialic acids. Recent human H3N2 viruses have lost the ability to bind 4-*O*-acetylated α2-3 linked sialic acids.

Supplementary Table 2: Compound map of the Glycan Microarray.

Locations of compound 1-27 are indicated (compound numbers refer to Figure 1). The compound code consists of “B” (biotin), 3 (2,3-linked), (2,6-linked) or 8 (2,8-linked), and 3 digits indicating the position of the acetylated hydroxyls with “0” for non-acetylated hydroxyls. “Buffer” is the printing buffer, “atto-biotir” is the biotin dye and “N’ is a gegative control (LacNAc).

B3000	B3000	B3000	B3000	B3000	B3000				B3479	B3479	B3479	B3479	B3479	B3479				B8079	B8079	B8079	B8079	B8079	B8079
B3009	B3009	B3009	B3009	B3009	B3009				B6000	B6000	B6000	B6000	B6000	B6000				B34N00	B34N00	B34N00	B34N00	B34N00	B34N00
B3400	B3400	B3400	B3400	B3400	B3400				B6400	B6400	B6400	B6400	B6400	B6400				B307N9	B307N9	B307N9	B307N9	B307N9	B307N9
B3070	B3070	B3070	B3070	B3070	B3070				B6070	B6070	B6070	B6070	B6070	B6070				B64N00	B64N00	B64N00	B64N00	B64N00	B64N00
B3079	B3079	B3079	B3079	B3079	B3079				B6009	B6009	B6009	B6009	B6009	B6009				B607N9	B607N9	B607N9	B607N9	B607N9	B607N9
B3000-2	B3000-2	B3000-2	B3000-2	B3000-2	B3000-2				B6409	B6409	B6409	B6409	B6409	B6409				N	N	N	N	N	N
B3400-2	B3400-2	B3400-2	B3400-2	B3400-2	B3400-2				B6079	B6079	B6079	B6079	B6079	B6079				buffer	buffer	buffer	buffer	buffer	buffer
B3070-2	B3070-2	B3070-2	B3070-2	B3070-2	B3070-2				B6479	B6479	B6479	B6479	B6479	B6479				atto-biotir	atto-biotir	atto-biotir	atto-biotir	atto-biotir	atto-biotir
B3009-2	B3009-2	B3009-2	B3009-2	B3009-2	B3009-2				B8000	B8000	B8000	B8000	B8000	B8000									
B3409	B3409	B3409	B3409	B3409	B3409				B8070	B8070	B8070	B8070	B8070	B8070									
B3079-2	B3079-2	B3079-2	B3079-2	B3079-2	B3079-2				B8009	B8009	B8009	B8009	B8009	B8009				atto-biotir	atto-biotir	atto-biotir	atto-biotir	atto-biotir	atto-biotir
