# Supplementary material for: Sialic acid O-acetylation patterns and glycosidic linkage type determination by ion mobility-mass spectrometry
Source: Nat Commun. 2023 Oct 25;14:6795. doi: 10.1038/s41467-023-42575-x (PMC10600165; doi:10.1038/s41467-023-42575-x)
Supplement: Supplementary file 2 — Reporting Summary [file 41467_2023_42575_MOESM2_ESM.pdf]

nature portfolio

Corresponding author(s): Boons, Geert-Jan; Sastre Toraño, Javier

Last updated by author(s): Sep 28, 2023

Reporting Summary

Nature Portfolio wishes to improve the reproducibility of the work that we publish. This form provides structure for consistency and transparency in reporting. For further information on Nature Portfolio policies, see our Editorial Policies and the Editorial Policy Checklist.

Statistics

For all statistical analyses, confirm that the following items are present in the figure legend, table legend, main text, or Methods section.

n/a	Confirmed	
☐	☒	The exact sample size (*n*) for each experimental group/condition, given as a discrete number and unit of measurement
☒	☐	A statement on whether measurements were taken from distinct samples or whether the same sample was measured repeatedly
☒	☐	The statistical test(s) used AND whether they are one- or two-sided<br>*Only common tests should be described solely by name; describe more complex techniques in the Methods section.*
☒	☐	A description of all covariates tested
☒	☐	A description of any assumptions or corrections, such as tests of normality and adjustment for multiple comparisons
☐	☒	A full description of the statistical parameters including central tendency (e.g. means) or other basic estimates (e.g. regression coefficient) AND variation (e.g. standard deviation) or associated estimates of uncertainty (e.g. confidence intervals)
☒	☐	For null hypothesis testing, the test statistic (e.g. *F*, *t*, *r*) with confidence intervals, effect sizes, degrees of freedom and *P* value noted<br>*Give P values as exact values whenever suitable.*
☒	☐	For Bayesian analysis, information on the choice of priors and Markov chain Monte Carlo settings
☒	☐	For hierarchical and complex designs, identification of the appropriate level for tests and full reporting of outcomes
☒	☐	Estimates of effect sizes (e.g. Cohen's *d*, Pearson's *r*), indicating how they were calculated

Our web collection on statistics for biologists contains articles on many of the points above.

Software and code

Policy information about availability of computer code

Data collection	LC-IM-MS data aquisition was performed using Agilent MassHunter Workstation Data Acquisition B.09 software.<br>Innopsys Mapix 8.1.0 was used to collect glycan microarray data.
Data analysis	M-MS:<br>Masses of raw 4 bit multiplexed IM-MS data were recalibrated on reference masses with m/z 121 and m/z 922 using the IM-MS data file reprocessing utility in the Agilent Technologies Masshunter software V10.0. Reprocessed IM-MS data was demultiplexed using the PNNL preprocessor software v4.0 (Pacific Northwest National Laboratory, Richland, WA) using an interpolation of 3 drift bins and a 5 point moving average smoothing. Features were identified with the Agilent Technologies Masshunter IMS browser software (v10.0) using an unbiased isotope model, allowing for single features with a maximum charge state of 5 and a minimal ion intensity of 500. High resolution ATDs were obtained using Agilent Technologies HRdm v2.0 software at processing level high, with an m/z width multiplier of 12, saturation check of 0.40 and an IF multiplier of 1.125 with SSS and post QC enabled.<br><br>Microarray data:<br>The data were processed with GenePix Pro 7 (Molecular Devices, San Jose, CA) and analyzed with an in-house developed Excel macro using Excel 2016 [https://github.com/enthalpyliu/carbohydrate-microarray-processing].

For manuscripts utilizing custom algorithms or software that are central to the research but not yet described in published literature, software must be made available to editors and reviewers. We strongly encourage code deposition in a community repository (e.g. GitHub). See the Nature Portfolio guidelines for submitting code & software for further information.

Data

Policy information about availability of data

All manuscripts must include a data availability statement. This statement should provide the following information, where applicable:

- Accession codes, unique identifiers, or web links for publicly available datasets
- A description of any restrictions on data availability
- For clinical datasets or third party data, please ensure that the statement adheres to our policy

The authors confirm that the data supporting the findings of this study are available within the article and/or its supplementary materials.

Data availability
The authors declare that the data supporting the findings of this study are available within the paper and its supplementary information files. The ion mobility-mass spectrometric source data that support the findings of this study are available in MassIVE (https://massive.ucsd.edu/ProteoSAFe/private-dataset.jsp?task=7053cb673b85441ba1e45e9e2d78645e) with the dataset identifier MSV000090864 [doi:10.25345/C57S7HX6G]. Glycan microarray source data are provided with this paper. The Excel Macro for batch processing of the glycan microarray data is availbale on the GitHub platform [https://github.com/enthalpyliu/carbohydrate-microarray-processing]. The sequence of HA ectodomain of equine H3 (A/Equine/Miami/1/1963 H3N8) is available at GenBank, accession no. AAA43105.1 (https://www.ncbi.nlm.nih.gov/search/all/?term=AAA43105.1).

Research involving human participants, their data, or biological material

Policy information about studies with human participants or human data. See also policy information about sex, gender (identity/presentation), and sexual orientation and race, ethnicity and racism.

Reporting on sex and gender	N/A
Reporting on race, ethnicity, or other socially relevant groupings	N/A
Population characteristics	N/A
Recruitment	N/A
Ethics oversight	N/A

Note that full information on the approval of the study protocol must also be provided in the manuscript.

Field-specific reporting

Please select the one below that is the best fit for your research. If you are not sure, read the appropriate sections before making your selection.

[x] Life sciences   [ ] Behavioural & social sciences   [ ] Ecological, evolutionary & environmental sciences

For a reference copy of the document with all sections, see nature.com/documents/nr-reporting-summary-flat.pdf

Life sciences study design

All studies must disclose on these points even when the disclosure is negative.

Sample size	No statistical methods were used to determine sample size since the study focused on method development using samples to demonstrate the applicability of the method. The selected number of experiments was chosen to reliably acquire results. Therefore, biologicals and six tissue samples were analyzed in one-fold with LC-IM-MS. LC-IM-MS experiments with synthetic standards were performed at technical triplicate and microarray experiments were performed at technical sextuplicate.
Data exclusions	Microarray experiments were performed at technical sextuplicate. As predetermined, the highest ans lowest replicates were removed to reduce the deviation of qualitative fluorescent data, and the mean and standard deviation were calculated (n=4).
Replication	Experiments with synthetic standards were performed at technical triplicate, proteins and tissue samples were analyzed in onefold and microarray experiments were performed at technical sextuplicate. All attempts at replication were succesfull.
Randomization	Samples were not randomized since the comparative analysis of the selected samples was not the goal of this study.
Blinding	Blindig was not applied since the comparative analysis of the selected samples was not the goal of this study.

Reporting for specific materials, systems and methods

We require information from authors about some types of materials, experimental systems and methods used in many studies. Here, indicate whether each material, system or method listed is relevant to your study. If you are not sure if a list item applies to your research, read the appropriate section before selecting a response.

Materials & experimental systems

n/a	Involved in the study	
☐	☒	Antibodies
☒	☐	Eukaryotic cell lines
☒	☐	Palaeontology and archaeology
☒	☐	Animals and other organisms
☒	☐	Clinical data
☒	☐	Dual use research of concern
☒	☐	Plants

Methods

n/a	Involved in the study	
☒	☐	ChIP-seq
☒	☐	Flow cytometry
☒	☐	MRI-based neuroimaging

Antibodies

Antibodies used	-Mouse anti-strep tag was made in-house, and goat-anti mouse-Alexa647 was obtained from Thermo Fisher Scientific (Waltham, MA, Ref#A21235 Lot#2306581).
Validation	The mouse anti-strep tag antibody was validated using an HA protein with either a strep tag or a his tag. The goat-anti mouse-Alexa647 antibody was validated by the supplier and reported online.
