# Supplementary material for: Sialic acid O-acetylation patterns and glycosidic linkage type determination by ion mobility-mass spectrometry
Source: Nat Commun. 2023 Oct 25;14:6795. doi: 10.1038/s41467-023-42575-x (PMC10600165; doi:10.1038/s41467-023-42575-x)
Supplement: Supplementary file 3 — Description of Additional Supplementary Files [file 41467_2023_42575_MOESM3_ESM.pdf]

Title: Supplementary Data 1
Description: Structure list of Endo-F2 released and procainamide labelled *N*-glycans identified in Myozyme. The structures are assigned according to enzymatic pathways and observations for structural assumptions of *N*-glycan structures as described in Supplementary Note 1 in the Supplementary Information.

Title: Supplementary Data 2
Description: Structure list of Endo-F2-released and procainamide-labelled *N*-glycans identified in Aflibercept. The structures are assigned according to enzymatic pathways and observations for structural assumptions of *N*-glycan structures as described in Supplementary Note 1 in the Supplementary Information.

Title: Supplementary Data 3
Description: List of PNGaseF released *N*-glycans identified in horse nasal tissue. The structures are assigned according to enzymatic pathways and observations for structural assumptions of N-glycan structures as described in Supplementary Note 1 in the Supplementary Information.

Title: Supplementary Data 4
Description: List of PNGaseF released *N*-glycans identified in horse nasal tissue, replicate 1. The structures are assigned according to enzymatic pathways and observations for structural assumptions of N-glycan structures as described in Supplementary Note 1 in the Supplementary Information.

Title: Supplementary Data 5
Description: List of PNGaseF released *N*-glycans identified in horse nasal tissue, replicate 2. The structures are assigned according to enzymatic pathways and observations for structural assumptions of N-glycan structures as described in Supplementary Note 1 in the Supplementary Information.

Title: Supplementary Data 6
Description: List of PNGaseF released *N*-glycans identified in horse frontal tracheal tissue. The structures are assigned according to enzymatic pathways and observations for structural assumptions of N-glycan structures as described in Supplementary Note 1 in the Supplementary Information.

Title: Supplementary Data 7
Description: List of PNGaseF released *N*-glycans identified in horse middle tracheal tissue. The structures are assigned according to enzymatic pathways and observations for structural assumptions of N-glycan structures as described in Supplementary Note 1 in the Supplementary Information.

Title: Supplementary Data 8
Description: List of PNGaseF released *N*-glycans identified in horse rear tracheal tissue. The structures are assigned according to enzymatic pathways and observations for structural assumptions of N-glycan structures as described in Supplementary Note 1 in the Supplementary Information.

Title: Supplementary Data 9
Description: Structure list of PNGAseF-released and procainamide-labelled N-glycans identified in equine α2-macroglobulin. The structures are assigned according to enzymatic pathways and observations for structural assumptions of N-glycan structures as described in Supplementary Note 1 in the Supplementary Information.

Title: Supplementary Data 10

Description: Structure list of neutralized hypochlorite released *O*-glycans identified in BSM. The structures are assigned according to enzymatic pathways and observations for structural assumptions of *N*-glycan structures as described in Supplementary Note 1 in the Supplementary Information.
